# Recombinant Complement Receptor 2 Radiolabeled with [^99m^Tc(CO)_3_]^+^ : A Potential New Radiopharmaceutical for Imaging Activated Complement

**DOI:** 10.1371/journal.pone.0018275

**Published:** 2011-04-06

**Authors:** Adam Badar, Sarah DeFreitas, James M. McDonnell, Norhakim Yahya, David Thakor, Reza Razavi, Richard Smith, Steven Sacks, Gregory E. D. Mullen

**Affiliations:** 1 Medical Research Council Centre for Transplantation, King's College London, Guy's Hospital, London, United Kingdom; 2 Division of Imaging Sciences, King's College London, St. Thomas' Hospital, London, United Kingdom; 3 Randall Division of Cell & Molecular Biophysics, New Hunt's House, King's College London, London, United Kingdom; Genentech, United States of America

## Abstract

We describe the design and synthesis of a new Tc-99m labeled bioconjugate for imaging activated complement, based on Short Consensus Repeats 1 and 2 of Complement Receptor 2 (CR2), the binding domain for C3d. To avoid non specific modification of CR2 and the potential for modifying lysine residues critical to the CR2/C3d contact surface, we engineered a new protein, recombinant CR2 (rCR2), to include the C-terminal sequence VFPLECHHHHHH, a hexahistidine tag (for site-specific radiolabeling with [^99m^Tc(CO)_3_(OH_2_)_3_]^+^). The protein was characterized by N-terminal sequencing, SDS-PAGE and size exclusion chromatography. To test the function of the recombinant CR2, binding to C3d was confirmed by enzyme-linked immunosorbent assay (ELISA). The function was further confirmed by binding of rCR2 to C3d^+^ red blood cells (RBC) which were generated by deposition of human or rat C3d and analyzed by fluorescence microscopy and flow cytometry. The affinity of rCR2 for C3d^+^, in presence of 150 mM NaCl, was measured using surface plasma resonance giving rise to a K_D_≈500 nM. Radiolabeling of rCR2 or an inactive mutant of rCR2 (K41E CR2) or an unrelated protein of a similar size (C2A) with [^99m^Tc(CO)_3_(OH_2_)_3_]^+^ at gave radiochemical yields >95%. Site-specifically radiolabeled rCR2 bound to C3d to C3d^+^ RBC. Binding of radiolabeled rCR2 to C3d was inhibited by anti-C3d and the radiolabeled inactive mutant K41E CR2 and C2A did not bind to C3d^+^ RBCs. We conclude that rCR2-Tc^99m^ has excellent radiolabeling, stability and C3d binding characteristics and warrants *in vivo* evaluation as an activated complement imaging agent.

## Introduction

Ischemia reperfusion injury (IRI) occurs when blood flow is restored to tissue after a period of ischemia [1]. It is responsible for much of the morbidity and mortality associated with conditions such as myocardial infarction, stroke, gut ischemia, and cardiopulmonary bypass. Ischemia reperfusion damage of transplanted organs such as kidney, heart, liver and lung also has a major impact on graft survival [2], [3], [4]. The pathophysiology of IRI is complex and consists of at least three major factors contributing to tissue injury: reactive oxygen species (ROS), white blood cells (neutrophils and macrophages), and components of the activated complement (C) cascade [5], [6], [7].

We have previously shown that complement proteins, such as C3, are synthesised by tissue parenchyma as an early response to tissue stress or infection [8], [9], [10]. C3, whether synthesized locally or deposited onto stressed cells by circulating C3, is cleaved on the target cell surface to form the C3b fragment and then further degraded into the membrane anchored C3dg and C3d fragments which remain covalently bound to the cell. The products of complement result not only in cell lysis but also opsonisation and recruitment of inflammatory cells. Complement activation has been described in the IRI of organs secondary to cardiovascular disease and in transplantation and can involve all three known pathways (the lectin (or mannose-binding) pathway, and the alternative and classical pathways). C3d is therefore a ‘footprint’ of complement activation. In several organ models of IRI, these terminal components of C3, C3b and C3d, are associated with tissue injury, and in transplanted organs with graft rejection [11], [12], [13]. Both C3 and C3b have a relatively short half-life in serum or on the membrane and C3b degrades within minutes on the plasma membrane of the effected cell. Thereafter C3d is relatively stable *in situ* [8]. Here we present the development of a Single Photon Emission Computed Tomography (SPECT) imaging agent that specifically binds to the degraded components of activated bound complement, namely C3dg and C3d, on the surface of affected cells. The endogenous human receptor of C3d is complement receptor type 2 (CR2 or CD21), and is comprised of 16 short consensus repeats (SCRs). The first two N-terminal domains, SCR1 and SCR2, are required for binding to C3d [14], [15] (Manuscript submitted, Supplemental Information). Here we describe a 15.5 kDa SCR1/2 fragment (recombinant CR2 or rCR2) which has been expressed, refolded and purified from E. Coli. The recombinant CR2 was expressed with an additional C-terminal histidine tag not only for ease of purification but also to allow site specific labeling with technetium [^99m^Tc(CO)_3_(OH_2_)_3_]^+^.

When radiolabeling small proteins such as these, it is often important to exert maximum control over the number and site of modifications to the molecule. Until recently nearly all molecular imaging studies have been performed using amine-reactive bifunctional agents. Generally, modification of the ε-amino groups of lysine within a protein is used for conjugation of radioisotopes via succinimide derivatives. Lysine modification has been the most common method of conjugation, but due to its high abundance in proteins there is an increased chance that a modification will occur in the binding region of the agent and render it inactive. In most cases the extent and statistical distribution of modification have not been measured, and it has not been quantitatively demonstrated that the bioconjugates retain full bioactivity compared with unmodified protein. In cases such as these, the detrimental effects are compounded because the lowest affinity (most heavily modified) protein molecules carry a disproportionately high radiolabel signal. Recently a study in which an anti-carcinoembrionic agent (CEA) diabody was labeled via lysines with N-succinimidyl 4-[18F] fluorobenzoate which led to an immunoreactivity of only 57% [16]. For this to be translated into the clinic the active agent would have to be purified to separate it from the inactive agent. We also recently demonstrated that non-specific “amine-directed” labeling of a cell death SPECT imaging agent, C2A (a domain of synaptotagmin I), led to abolition of binding to its target, phosphatidylserine, while the inclusion of a novel site specific C-terminal hexahistadine tag fully retained function [17].

Given these problems, producing recombinant protein conjugates to current good manufacturing practice (cGMP) standards with consistent batch-to-batch quality using non-site-specific modification for clinical imaging is problematic. Removal of any inactive or low affinity protein prior to injection, if achievable at all, requires additional affinity chromatography purification steps. A cGMP recombinant protein imaging agent developed for clinical application should be labeled site-specifically, reproducibly, efficiently and at room temperature. Preferably it should be a simple kit-based method providing a well-characterized, homogeneous, fully functional and stable product.

With this in mind, we have genetically engineered rCR2 with a C-terminal domain designed for site-specific modification with maximum versatility, incorporating a HexaHistidine tag (His-tag). Here, the term site-specific refers to the ability to radiolabel a radiopharmaceutical via His-tag without interfering with its function. The His-tag can be used not only for immobilized metal affinity chromatography (IMAC) purification, but also in principle for site-specifically labeling with ^99m^Tc using [^99m^Tc(CO)_3_(OH_2_)_3_]^+^ [18]. The His-tag is non-immunogenic and does not hinder the clinical development of His-tag -containing recombinant proteins [19], [20], [21], [22]. In this paper we report the use of this strategy to develop a site-specific radiopharmaceutical for the imaging of activated complement.

## Materials and Methods

### Molecular Biology

The recombinant Complement Receptor 2 (rCR2) gene was designed using the amino acid residues 21–154 of human CR2 (Swiss-Prot accession: P20023 [23]) containing the Short Consensus Repeats (SCR) one and two, the linker region between SCR two and SCR three with the addition of a C-terminal tag including the SCR2 to SCR3 linker (VFPLEC) and a hexahistidine tag. The synthetic rCR2 DNA was codon optimized for bacterial expression, synthesized, and sub-cloned into the pET-24a vector (Merck Chemicals, UK) using the restriction enzymes NdeI and EcoRI, by GeneArt (Regensburg, Germany). The rCR2 gene was sequenced by King's College London, Molecular Biology Unit, UK.

After sequence confirmation the rCR2 containing plasmid was transformed into the BL21 (DE3) (Invitrogen, UK) strain of *Escherichia coli* and selected on kanamycin (50 µg/mL) agar plates. An individual colony was selected to inoculate a seed culture in 10 mL Luria Bertani (LB) media containing 50 µg/mL kanamycin (Sigma, Gillingham, UK) and grown overnight at 37°C with agitation. The seed culture was then used to inoculate 500 mL LB media and grown at 37°C in baffled flasks with agitation until the OD_600_ reached 0.7. Expression was then induced by addition of 1 mM isopropyl β-D-1-thiogalactopyranoside (IPTG) (Merck Chemicals, Nottingham, UK) followed by further incubation at 37°C with agitation for 4 h. Cells were then pelleted by centrifugation at 4,100 relative centrifugal force (RCF) for 10 min. and the cell pellets stored at −80°C. Frozen pellets were thawed and resuspended in 20 mL sonication buffer, 50 mM Tris-HCl (Fisher Scientific, Loughborough, UK ) pH 8.0, 150 mM NaCl (Sigma, Gillingham, UK), 1 mM EDTA (Fisher Scientific, Loughborough, UK), 0.1% TritonX-100 (Alfa Aesar, Heysham, UK) and Complete Protease Inhibitors (Roche Diagnostic, Burgess Hill, UK) and lysed via sonication at 60% amplitude on ice for 5 bursts of 15 sec. with 30 sec intervals. The suspension was then centrifuged at 4°C for 20 min. at 35,000 RCF.

A SDS PAGE gel was transferred onto PVDF membrane (Invitrogen, UK) and the protein bands excised and analyzed via N-terminal sequencing (Department of Biochemistry, University of Cambridge).

A protein refolding procedure was adapted from the refolding of SCR1–3 of the human CR1 [24]. Briefly, the insoluble fraction, containing mostly inclusion bodies, was washed 3 times with 35 ml of wash buffer (50 mM Tris HCl pH 8.0, 1 mM EDTA, 50 mM NaCl, complete protease inhibitors), resuspending the pellet and centrifugation at 35,000 RCF for 20 min. The washed inclusion bodies were then immediately solubilised by slowly adding 150 mL of solubilising buffer (8 M urea, 20 mM Tris HCl pH 8.5, 1 mM EDTA, 5 mM DTT, protease inhibitors, pH 8.5) while stirring. The solution was then gently mixed at room temperature (RT) for 2 hrs. The inclusion body solution was then slowly pumped at 1 mL/min. into 4 L of refold buffer (300 mM ethanolamine (Sigma, Poole, UK), 1 mM EDTA, 1 mM cysteine (Sigma, Poole, UK), 2 mM cystine (Sigma, Poole, UK), protease inhibitors, pH 11, prepared the day before and cooled to 4°C) while gently stirring. The sample was then stored at 4°C for 24 hours for refolding to occur. The 4 L of refold buffer containing the rCR2 was concentrated to ∼50 mL by ultrafiltration using a Vivaflow 200 5 kDa membrane cutoff tangential flow filtration system (Sartorius Stedim, Surrey, UK). The sample was recirculated through the unit using a peristaltic pump at ∼200 mL/min., at a pressure of ∼2.5 bar. In preparation for immobilized metal affinity chromatography, the protein was dialysed overnight into nickel binding buffer (20 mM sodium phosphate pH 7.4, 0.5 M NaCl, 40 mM Imidazole) After dialysis the solution was added to a 1 mL nickel column (GE Healthcare, Amersham, UK), which had previously been equilibrated with nickel binding buffer, at 1 mL/min. using an AKTA FPLC (GE Healthcare, Amersham, UK). Impurities were washed with 100 mM imidazole and rCR2 was eluted with 400 mM imidazole). 0.5 mL fractions were collected and analysed by SDS PAGE. 3 mL of eluate from the IMAC column was then loaded on a Sephacryl 100 size exclusion column (60 cm height and 2.6 cm diameter, GE Healthcare, Amersham, UK), which had been equilibrated with size exclusion buffer (PBS pH 7.4 and 0.5 M NaCl), at a flow rate of 1 mL/min. 1 mL fractions were collected and analyzed by SDS PAGE.

The mutation of lysine 41 to glutamic acid in SCR 1 of CR2 (K41E CR2) has previously been shown to abrogate binding to C3d. It was expressed, purified and characterised as previously described (Manuscript submitted, Supplemental information).

### rCR2 ELISA protocol

Measurement of rCR2 binding to human C3d was performed according to a standardized ELISA protocol. 96-well ELISA plates (Greiner-one high protein binding, Sigma UK) were coated with C3d protein (Merck, Darmstadt, Germany) at increasing two fold concentrations ranging from 1.5 to 1500 nM/well and incubated at 4°C overnight in PBS pH 7.4. Plates were then washed three times with wash buffer (1% Tween-PBS, pH 7.4) and blocked at RT for 1 hr with blocking buffer (3% Bovine Serum Albumin (BSA, Sigma, UK) in PBS pH 7.4). The plate was then washed 3 times with wash buffer. rCR2, or C2A (domain A of synaptotagmin I [17]), which is an irrelevant His-tagged protein of the same size, were diluted in 0.05% BSA-PBS pH 7.4, added to antigen coated wells in triplicate at a fixed concentration of 1.5 µM per well, and incubated for 1 h. at room temperature. The plate was washed three times with wash buffer and plates were incubated with anti-His secondary antibody (Quiagen,UK) at a dilution of 1∶1000 in 0.05% BSA-PBS pH 7.4 for 1 h. This was followed by a further washing step and then addition of the substrate o-phenylenediamine (OPD) at 1 mg/mL and adding 2 µl of 30% H_2_O_2_. After 30 minutes the absorbance at 450 nm was read using a SPECTRAmax 340PC microplate reader (Molecular Devices Co., Sunnyvale, CA). On each ELISA plate three control wells were included in triplicate where no C3d, rCR2 or secondary antibody had been added. The assay was performed in triplicate and at least 3 independent experiments were performed.

### Surface Plasmon Resonance

Binding of rCR2 to C3d was carried out on a Biacore T100 system (GE, Healthcare, Amersham, UK) at 25°C, according to the manufacturer's guidelines. Recombinant CR2 was dialysed into running buffer (0.01 M HEPES, pH 7.4, 0.15 M NaCl, 3 mM EDTA, 0.005% Tween 20). A C3d construct, engineered to include a free exposed thiol group, was generated by reversing the initial Cys17Ala mutation (corresponding to Cys1010 of full length C3). The resulting protein was immobilized onto a CM5 sensor chip by thiol coupling of the free Cys17 using a ligand thiol coupling kit according to the manufacturer's instructions (Biacore AB). A flow rate of 30 µl/min. was used and the ligand contact time was 60 seconds with a dissociation time of 1200 seconds. Analyte (rCR2) concentrations were injected in duplicate, involving a titration series of “low to high” analyte concentration followed by a “high to low” series [25]. Buffer blanks were also included in the titration series. A blank flow cell was used as a control surface. SPR data was processed with the Biaevalution software 3.1 (Biacore AB). Curve fitting and other analyses were performed using MicroCal Origin v7 (OriginLab).

### C3d^+^ Red blood Cell binding assay

To generate C3d positive red blood cells (C3d^+^ RBC) in the absence of cell lysis, 1×10^8^ sensitized sheep RBC (Diamedix, Rome, Italy) in 3 mL of gelatin veronal buffer (Sigma) were treated with either 25 µl of rat C6 deficient serum (kindly provided by Professor Frank Baas, University of Amsterdam, Netherlands) or human C6 deficient serum (Sigma) for 30 min. at 37°C. Confirmation of C3d deposition onto the RBC membrane was confirmed by incubating C3d^+^ RBC with a primary anti-human C3d at a dilution of 1∶100 followed by a wash and incubation with a secondary mouse anti-human-FITC at a dilution of 1∶200. Cells were then analysed by immunofluorescent microscopy (Olympus Bx51, Southend-on-Sea, UK) on a glass cover slide and by flow cytometry (FACScan, Becton Dickinson, Oxford UK). To test the binding capability of rCR2 to native human C3d, 1.5 µM rCR2, C2A or K41E CR2 were each incubated with 2.5×10^7^ C3d^+^ RBCs in 1.5 mL of PBS and 2 mM EDTA for 60 min. The cells were then centrifuged at 360 RCF, washed 3 times with PBS and then incubated with a secondary anti-HIS-FITC (Abcam, Cambridge, UK). The assay was performed in triplicate and repeated in three independent experiments. Cells were then analysed by immunofluorescent microscopy and a flow cytometer. To control for non-specific binding unstained cells and IgG1 K isotype antibody (eBiosciences, Hatfield, UK) were also tested. To further determine the specificity of rCR2 for C3d, 2.5×10^7^ C3d^+^ RBC were pre-incubated with 10 µg/mL of anti-human C3d prior to rCR2 and secondary anti-HIS-FITC incubation.

### ^99m^Tc labeling of rCR2, K41E CR2 and C2A

^99m^Tc pertechnetate eluted with saline from a Drytec generator (GE Healthcare, Amersham, UK) was converted to [^99m^Tc(CO)_3_(OH_2_)_3_]^+^ using an adapted kit from Isolink kit® (generously provided by Covidien, Petten, The Netherlands). Quality control was carried out according to manufacturer's instructions using instant thin layer chromatography (ITLC) and analysis with a gamma-ray TLC scanner (Lablogic, UK) and high performance liquid chromatography (HPLC series 1200, Agilent, UK) equipped with an in-line gamma detector (Lablogic, UK). Proteins were labeled with ^99m^Tc by incubating 100 µg of rCR2, C2A or K41E CR2 in 100 µL of PBS pH 7.4 with an additional 350 mM NaCl and up to 1 GBq of [^99m^Tc(CO)_3_(OH_2_)_3_]^+^ in 100 µL at 37 C°. Unless otherwise stated the labeling of all species was performed at a specific activity of 4 MBq/µg. However, to reproduce a high (clinically relevant) specific activity, rCR2 was also labeled at 10 MBq/µg. Radiolabeling efficiency was determined by ITLC as described above and the radiolabeling efficiency was calculated as a ratio of the radiolabeled protein peak integral (R_f_ = 0) to the unincorporated [^99m^Tc(CO)_3_(OH_2_)_3_]^+^ peak integral (R_f_ = 1.0).

### Binding of ^99m^Tc-rCR2 to C3d^+^ cells

Binding of ^99m^Tc-rCR2, K41E ^99m^Tc-rCR2 and ^99m^Tc-C2A to C3d was evaluated by a C3d^+^ red blood cell assay as described above. In the C3d^+^ red blood cell assay, 2.5×10^7^ C3d^+^ RBCs and C3d- RBCs were incubated with ^99m^Tc-rCR2, K41E ^99m^Tc-CR2, ^99m^Tc-C2A or 10 µg/mL anti-human C3d followed by ^99m^Tc-rCR2. Cells were then centrifuged at 360 RCF and washed five times with PBS. The percent of total radioactivity bound to C3d^+^ RBCs was determined as a ratio of radioactivity associated with the cell pellet, as determined by gamma counting (Wallac, 1282 COMPUGAMMA, PerkinElmer, UK), vs. total radioactivity added. The assay was performed in triplicate and repeated in three independent experiments.

### Binding of ^99m^Tc-rCR2to C3d

Binding of ^99m^Tc-rCR2 to C3d was evaluated by ELISA using a similar protocol as described above. However, ELISA 12 well strips (Greiner Bio-One Ltd, Stonehouse, UK) were used which allowed for individual wells to be separated and gamma counted. ^99m^Tc-rCR2 was incubated in triplicate and the bound ^99m^Tc-rCR2 was determined by gamma counting the individual ELISA wells. The radioactive ELISA was repeated in three independent experiments. The saturation binding data was analyzed with nonlinear regression assuming the one site specific binding model using the equation Y = B_max_*X/(K_d_+X) using GraphPad Prism (GraphPad Software Inc. San Diego, USA).

### Serum stability

1 mg/mL rCR2 and K41E CR2 were labeled with 400 MBq [^99m^Tc(CO)_3_(OH_2_)_3_]^+^ for 30 min. at 37°C with gentle shaking. After purification on a vivaspin500 (MW 3000 kDa Sartorius, UK), labeled rCR2 or K41E CR2 was added to human serum (Sigma) at 1∶10 v/v. As a control, labeled rCR2 or K41E CR2 was also added to PBS at 1∶10 v/v. The samples were then incubated at 37°C. At 0.5, 1, 2, 4, 8 and 24 h. samples were taken and analyzed by instant thin layer chromatography (ITLC) using a mobile phase of methanol and 1% concentrated HCl. As a control a separate ITLC of [^99m^Tc(CO)_3_(OH_2_)_3_]^+^ and [TcO_4_]^−^ was performed in the same mobile phase. ITLC were then monitored using a radio TLC scanner (LabLogic, UK). Serum stability was calculated as the area under the protein peak (Rf = 0) versus the area under the curve of the remainder of the chromatogram ([^99m^Tc(CO)_3_(OH_2_)_3_]^+^ or [TcO_4_]^−^ Rf = 1.0).

## Results

### Cloning, preparation and characterization of rCR2

**Figure 1:**
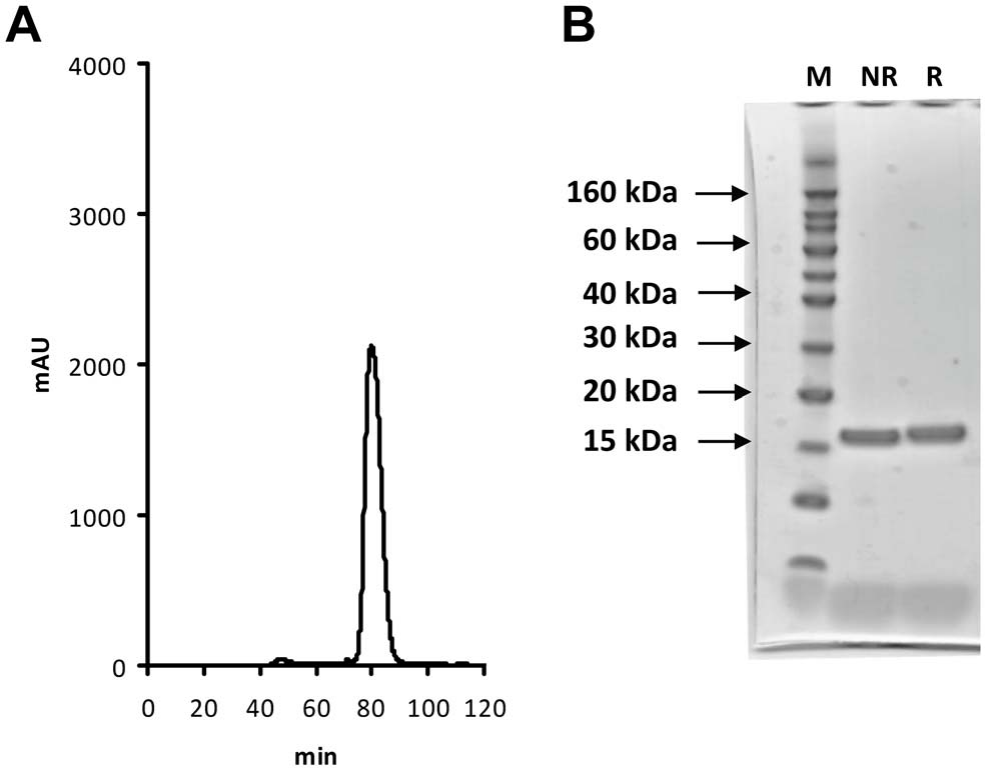
Size exclusion, SDS/PAGE and ES-MS characterization of rCR2. (***A***)After expression and purification by IMAC, rCR2 was further purified by preparative size exclusion chromatography. In the fast protein liquid chromatography (FPLC) size exclusion chromatogram shown, a discrete single peak was observed with a retention time of 80 min. as expected for a globular ∼15 kDa protein compared to molecular weight standards (data not shown). (***B***) The single peak obtained from the preparative size exclusion chromatography was analyzed by non-reduced (NR) and reduced (R) SDS/PAGE electrophoresis. In the SDS PAGE gel shown, a single band under both the non reduced and reduced conditions of ∼15 kDa was observed as compared to molecular weight markers (M). This indicates that rCR2 exists as a single species and no dimer is present.

The first two short consensus repeats (SCR) of complement receptor 2 (CR2) were cloned into the pET24a bacterial expression vector with the addition of a C-terminal spacer and a HexaHistidine tag (His-tag), forming the construct rCR2. After induced expression in *E. coli*, rCR2 was isolated from the insoluble fraction via an inclusion body preparation, solubilised and refolded by high dilution method in a redox buffer. Recombinant CR2 was purified first via IMAC and further purified by size exclusion chromatography (Figure 1a). Recombinant CR2 eluted as a single discrete peak with a retention time expected for a protein of ∼16 kDa, with no significant evidence of aggregation or dimerization. Recombinant CR2 was analyzed by reduced and non-reduced SDS PAGE, giving rise to a single monomeric band at the expected molecular size (Figure 1b). When samples were stored for prolonged periods and exposed to air, the formation of a dimer was apparent by SDS PAGE (data not shown). The final protein product was analyzed by N-terminal sequencing of the first ten amino acids, giving the expected sequence of ISCGSPPPIL.

### Recombinant CR2 binds to immobilized C3d

**Figure 2:**
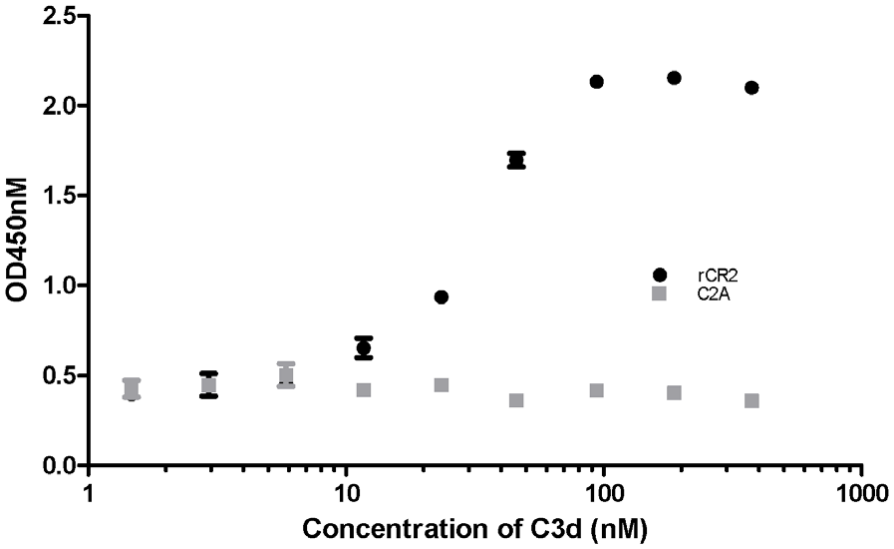
rCR2 binding to C3d as measured by ELISA. To determine the binding of rCR2 to C3d, the antigen C3d was coated on ELISA plates at increasing two fold concentrations. The ELISA wells were then incubated with rCR2 (circles) and an irrelevant protein C2A (squares) followed by anti-HIS HRP and o-phenylenediamine substrate. Control ELISA wells with no C3d antigen or no rCR2 or anti-HIS HRP alone gave optical densities similar to background (data not shown). Error bars represent the standard deviation of the mean of the triplicate.

*In vitro* binding of rCR2 to C3d was confirmed by ELISA and binding to C3d^+^ RBCs which was confirmed by immunofluorescent microscopy and flow cytometry. An rCR2 ELISA was developed to test binding to immobilized C3d. Human C3d was plated at increasing concentrations and then incubated with rCR2 followed by incubation with anti-HIS-HRP and substrate. rCR2 bound to C3d and showed an EC_50_ (estimated concentration that corresponds to 50% of the OD_450_ response range) of ∼48.5 nM (Figure 2). Recombinant CR2 did not bind to empty ELISA wells, and anti-HIS-HRP did not bind to C3d, both were comparable to background absorptions. An irrelevant His-tag protein of similar molecular weight, C2A, was tested in the ELISA and showed no significant binding to C3d.

**Figure 3:**
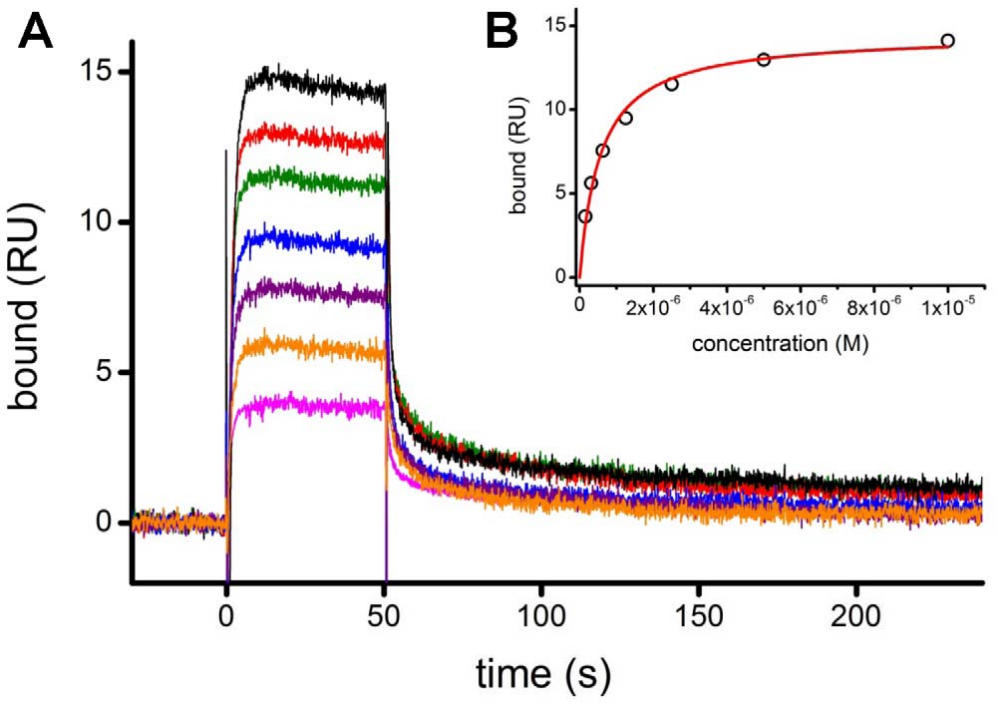
Surface plasmon resonance of rCR2. (***A***) A representative SPR sensorgram of rCR2 binding to immobilized C3d. C3d was coupled to the SPR sensor chip using the free thiol of the Cys17 residue. Binding was determined over a range of rCR2 concentrations; shown here, 78 (pink), 156 (orange), 313 (purple), 625 (blue), 1250 (green), 2500 (green), 5000 (red) and 10000 nM (black). (***B***) The binding affinity of rCR2 to C3d was determined by plotting the binding response at equilibrium against the concentration of rCR2 and fitting a Langmuir binding isotherm.

To determine the binding affinity of rCR2 to C3d, surface plasmon resonance (SPR) analysis was performed. To simulate native binding conditions a C3d construct that possessed a cysteine residue at position 17 was used. In native C3d is modified to produce a buried thioester moiety with Gln20. The acyl-amidazole activated side chain can then form a covalent bond with nucleophiles during C3d-antigen interactions [26]. The free thiol of Cys17 was used to immobilize C3d to the carboxymethylated surface of the SPR chip, ensuring an oriented, homogeneous immobilized C3d, with its ligand binding site exposed for interaction with CR2. The rCR2/C3d interaction displays “fast-on/fast-off” kinetics, where the association phase reaches equilibrium and the bound complex dissociates at least as fast as the time required to transfer new buffer into the SPR flowcell (Figure 3a). “Fast-on/fast-off” kinetics makes equilibrium analysis straightforward but prevents precise measurement of association and dissociation rate constants.

The binding affinity was determined by plotting the binding response at equilibrium against the concentration of rCR2 and fitting a Langmuir binding isotherm (Figure 3b). The Langmuir model assumes that both binding partners are homogenous, the analyte is monovalent, and that all binding events are independent [27].This analysis indicates a K_D_ of 570±30 nM and the observed Bmax value is consistent with a 1∶1 binding stoichiometry; these binding characteristics, in the presence of 150 mM NaCl, are in agreement with ones previously reported for this interaction [28]. The non-binding mutant K41E rCR2 has previously been tested in this system and also showed no binding in the SPR experiment (manuscript submitted).

**Figure 4:**
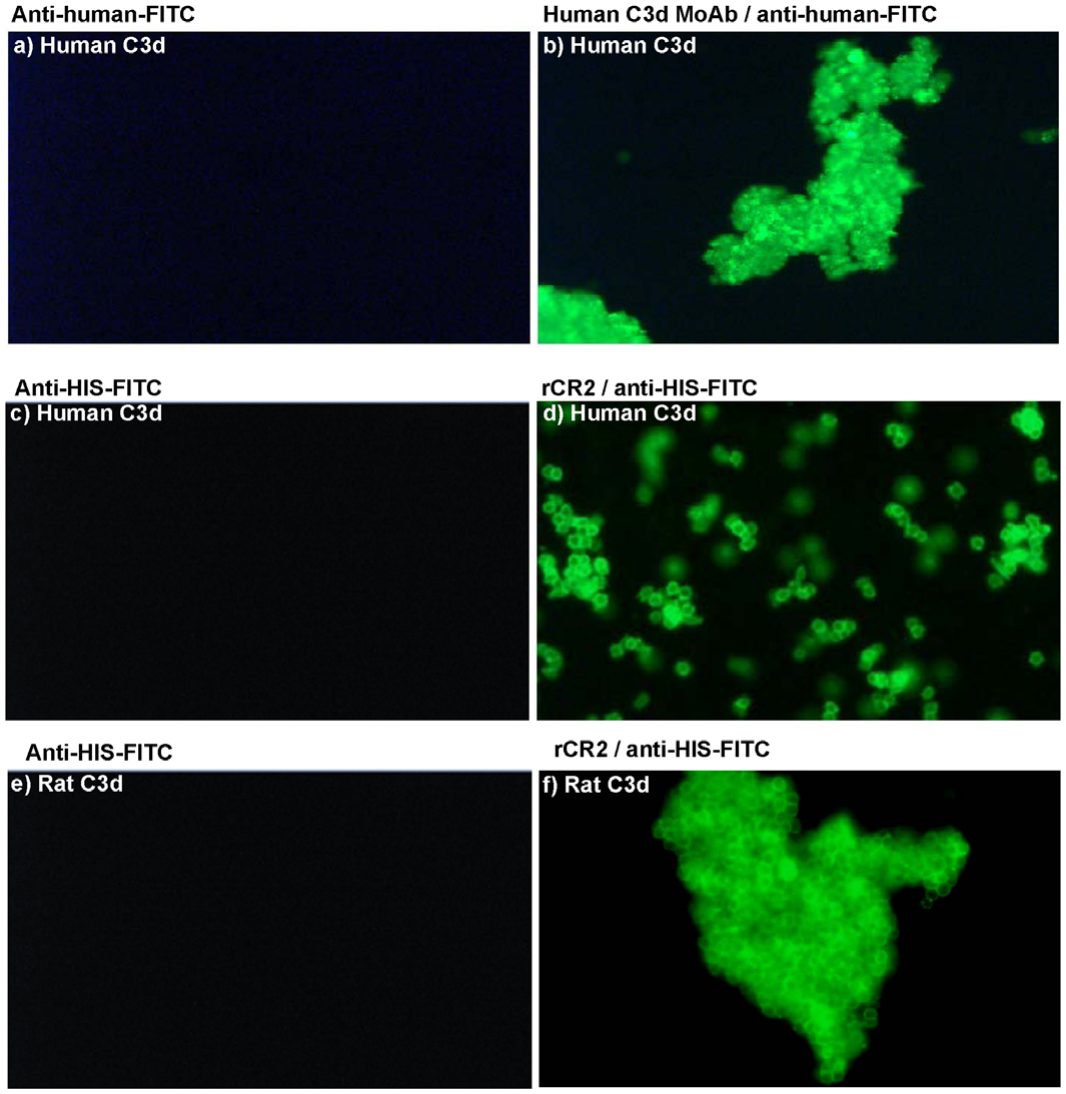
Immunofluorescent microscopy of rCR2 or anti-C3d bound to C3d^+^ red blood cells. Sensitised sheep red blood cells were incubated with either human (**panels a, b, c and d**) or rat (**panels e and f**) C6 deficient serum to generate C3d plasma membrane deposition in the absence of cell lysis. Cells were then incubated with either anti-human C3d followed by mouse anti-human-FITC secondary (**panel b**) or secondary antibody only (**panel a**). C3d^+^ cells were also incubated with rCR2 followed by secondary anti-HIS-FITC (panel d and f) or secondary alone (**panel c and e**).

The ability of rCR2 to bind to C3d was also monitored by the incubation of rCR2 with sensitized sheep red blood cells which had previously been treated with C6 deficient human or rat serum to induce the deposition of human or rat C3d, respectively. The confirmation of human C3d deposition was enabled by immunofluorescent microcopy using an anti-human C3d antibody (Figure 4b). The binding of rCR2 was confirmed by incubating human or rat C3d^+^ RBCs with rCR2 followed by staining with anti-HIS-FITC (Figure 4d and 4f). Specificity of rCR2 binding to C3d was confirmed by the absence of the K41E CR2 mutant binding to C3d^+^ RBCs (Data not shown).

**Figure 5:**
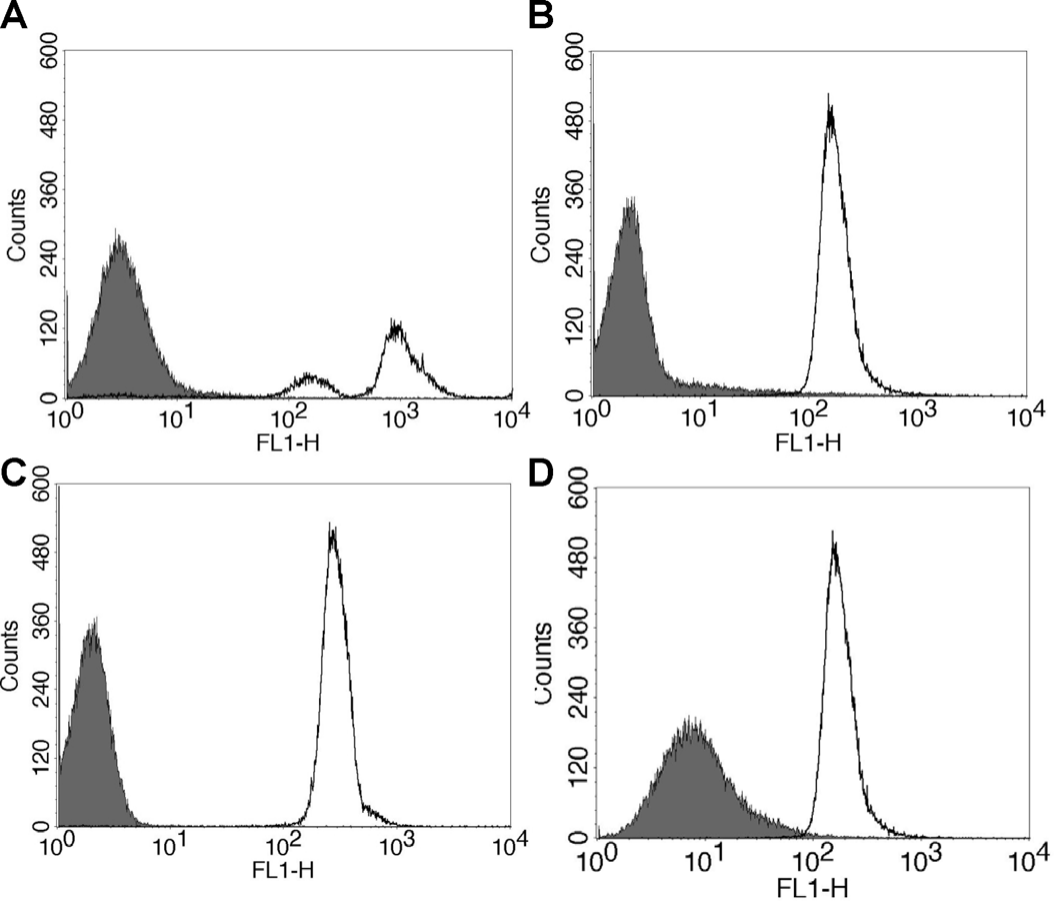
Flow Cytometry of rCR2 or anti-C3d bound to C3d^+^ red blood cells. Sensitised sheep red blood cells were incubated with human C6 deficient serum to generate C3d deposition on RBC in the absence of cell lysis. (***A***) To confirm C3d deposition, C3d^+^ RBC incubated with anti-human C3d (black line) or isotype control (solid grey) followed by mouse anti-human-FITC secondary. (***B***) To demonstrate rCR2 binding to C3d, C3d^+^ RBC cells were incubated with rCR2 (black line) or an irrelevant His-tag protein, C2A, (solid grey) followed by secondary anti-HIS-FITC. (***C***) To further demonstrate that the mutant K41E CR2 mutant did not bind to C3d, C3d^+^ RBC cells were incubated with rCR2 (black line) or K41E CR2 (solid grey) followed by secondary anti-HIS-FITC. (***D***) To demonstrate that rCR2 specifically bound to C3d, C3d^+^ RBC cells were first incubated with the inhibitory anti-human C3d prior to incubation with rCR2 and secondary anti-HIS-FITC (solid grey) or as positive control rCR2 and secondary anti-HIS-FITC alone (black line).

Binding of rCR2 to C3d^+^ cells was also evaluated by flow cytometry, whereby human C3d^+^ RBCs were generated by exposing sensitized sheep red blood cells to C6 deficient human sera. C3d deposition was confirmed by using an anti-human C3d antibody followed by an anti-human FITC secondary. Specificity of the antibody was confirmed using C3d^−^ RBCs and incubation with secondary antibody alone (Figure 5a) which showed no binding to RBCs. Confirmation of rCR2 binding to human C3d^+^ cells was demonstrated by incubating the cells with rCR2 followed by secondary anti-HIS-FITC (Figure 5b). As a control experiment an irrelevant His-tag protein, C2A, was incubated with C3d^+^ RBCs followed by secondary anti-His-FITC and showed no binding to C3d^+^ cells (Figure 5c). The specificity of the rCR2 was corroborated by the absence of immunostaining following blockade of C3d with anti-C3d blocking antibody(Figure 5d), secondary antibody only and incubation with C3d- RBCs (Figure 5b), all which showed no binding to RBCs. The specificity was further corroborated when K41E CR2 mutant showed no binding to C3d^+^ RBCs.

### Labeling of rCR2 and K41E CR2 with [^99m^Tc(CO)_3_]^+^

**Figure 6:**
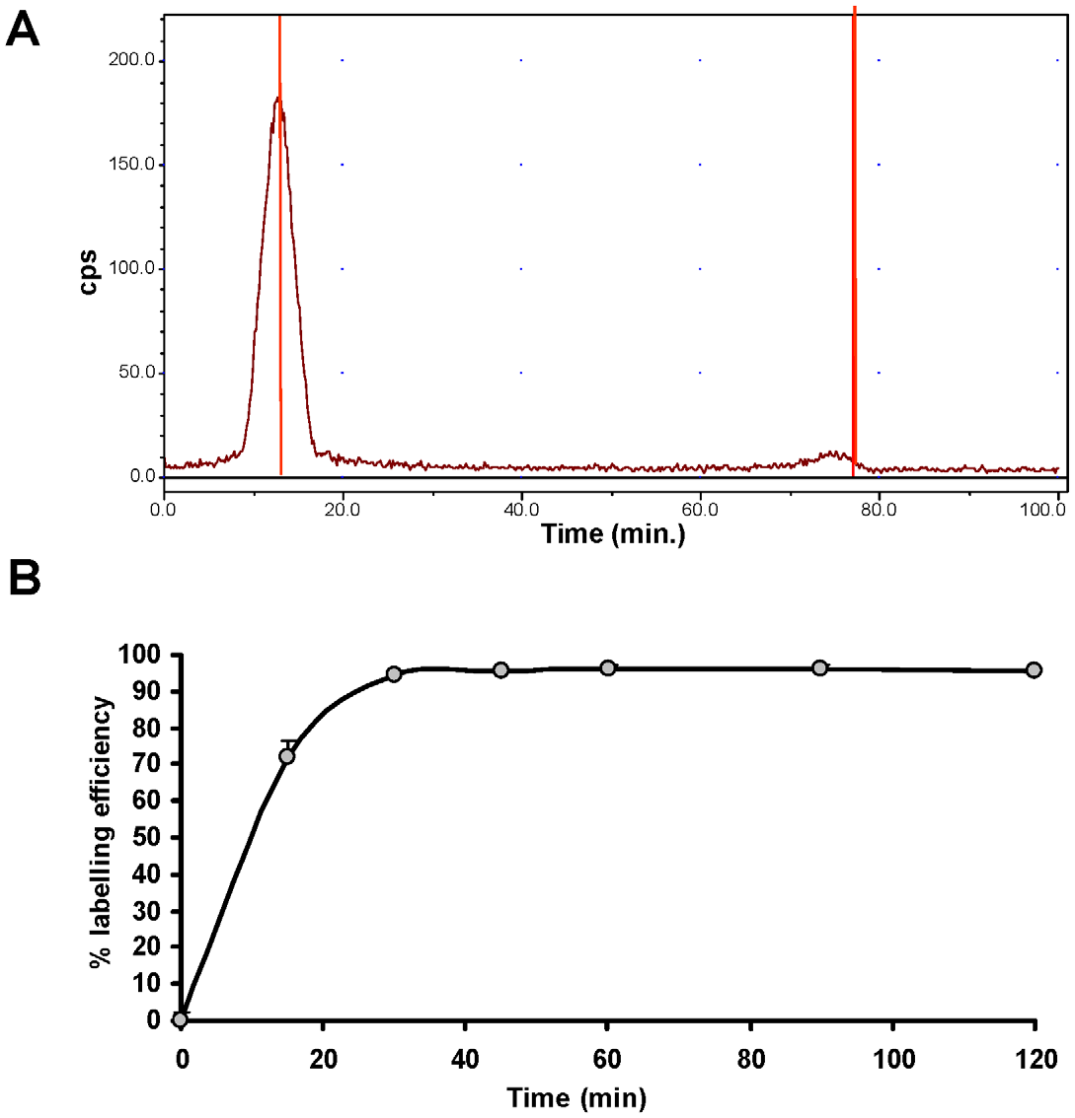
Radiolabeling of rCR2. (***A***) rCR2 was incubated with [^99m^Tc(CO)3(OH2)3]^+^ at 37°C for 30 minutes at a protein concentration of 1 µg/µL and analysed by instant thin layer chromatography (ITLC). The radiolabeling efficiency was calculated as a ratio of the radiolabeled protein peak integral (R_f_ = 0) to the unincorporated [^99m^Tc(CO)_3_(OH_2_)_3_]^+^ peak integral (R_f_ = 1.0). (***B***) The radiolabeling efficiency was followed over a 120 minute period. Aliquots were removed periodically and analysed by ITLC and the radiolabeling efficiency was determined over time.

Recombinant CR2 and K41E CR2 mutant were labeled at 1 mg/mL by incubating [^99m^Tc(CO)_3_(OH_2_)_3_]^+^ with 100 µg of protein at 37°C with gentle shaking for up to 120 min. Radiochemical yield was determined by ITLC (Figure 5a). A radiochemical yield of >95% was achieved with 1 µg/µL of rCR2 and K41E CR2 within 30 min at 37°C. Longer incubation times did not significantly improve the radiochemical yield (Figure 6b). When rCR2 was radiolabeled at higher and more clinically relevant specific activities (i.e. 7 MBq/µg), at 37°C for 30 min., radiolabeling efficiency of 95% was achieved.

### Recombinant ^99m^Tc-CR2 binds to C3d

**Figure 7:**
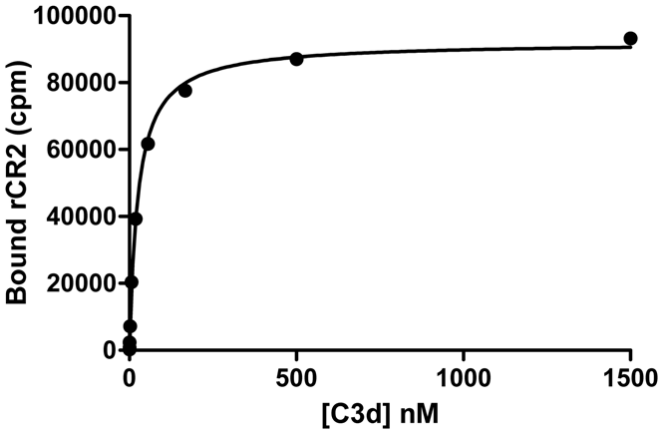
^99m^Tc-rCR2 binds to C3d. To determine the binding of ^99m^Tc-rCR2 to C3d, C3d antigen was coated on ELISA plates at increasing two fold concentrations. The ELISA wells were then incubated with rCR2. The supernatant was removed and after washing the individual ELISA wells were gamma counted. The saturation binding data was analysed using a non linear regression model assuming a one site specific binding using the equation Y = B_max_*X/(K_d_+X). Error bars represent the standard error of the mean of the triplicate.

**Figure 8:**
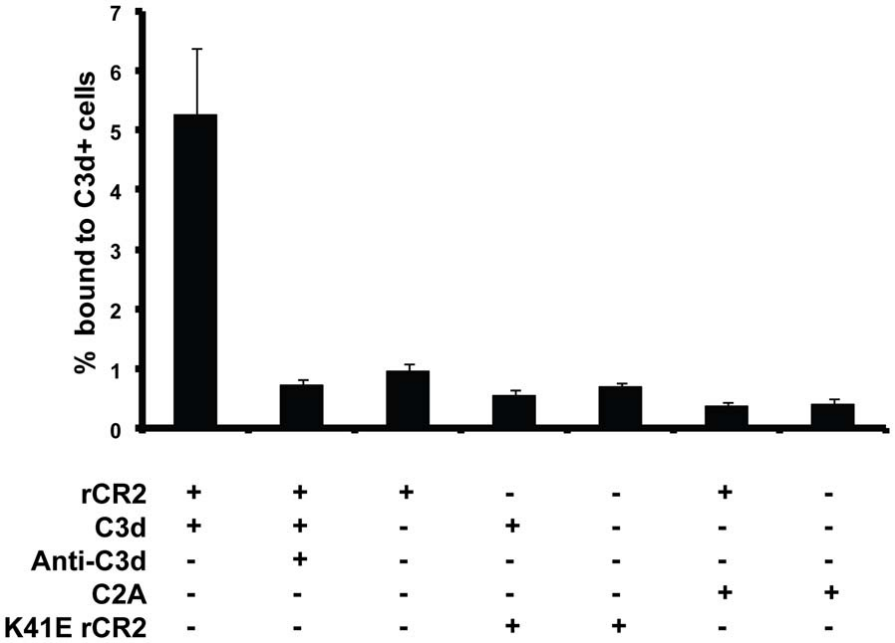
^99m^Tc-rCR2 binds to C3d^+^ RBC. Sensitised sheep red blood cells were incubated with human C6 deficient serum to generate C3d deposition on RBC in the absence of cell lysis. ^99m^Tc-rCR2 , ^99m^Tc-C2A or K41E ^99m^Tc-rCR2 were added to 2.5×10^7^ C3d^+^ cells. The percentage radioactivity bound to C3d^+^ cells was determined by gamma counting the activity associated with the cell pellet as percentage of total activity added. Error bars represent the standard error of the mean of n = 5.

*In vitro* binding of ^99m^Tc-rCR2 to C3d was confirmed by ELISA and binding to C3d^+^ RBCs. The ELISA was used to test ^99m^Tc-rCR2 binding to immobilized C3d and demonstrated that ^99m^Tc-rCR2 bound to C3d but did not bind in the absence of C3d (data not shown). Using a non linear regression model to fit the saturation binding curve gave rise to a B_max_ 92067 (SE ±1143) and a K_d_ = 25.47 nM (SE ±1.48 nM) (Figure 7). Binding of ^99m^Tc-rCR2 to C3d^+^ cells was also evaluated in a modified C3d RBC assay as described above. Confirmation of ^99m^Tc-rCR2 binding to human C3d^+^ cells was demonstrated by incubating the C3d^+^ RBCs with ^99m^Tc-rCR2 and measuring the radioactivity associated with the cells (Figure 7). As control conditions ^99m^Tc-K41E CR2 and ^99m^Tc-C2A were also incubated with C3d^+^ RBCs and showed no binding to C3d^+^ cells (Figure 8). The specificity of the ^99m^Tc-rCR2 was further corroborated by the absence of binding to C3d^+^ RBCs with C3d antibody inhibition (Figure 8).

### Serum stability of ^99m^Tc radiolabeled rCR2 and K41E CR2

The stabilities of rCR2-[^99m^Tc(CO)_3_] and K41E CR2-[^99m^Tc(CO)_3_] during incubation in PBS or serum over 24 h were determined using ITLC with gamma detection (Table 1). ^99m^Tc-rCR2, K41E ^99m^Tc-CR2, [^99m^TcO_4_]^−^ and [^99m^Tc(CO)_3_(OH_2_)_3_]^+^ were analyzed beforehand in order to determine their migration on ITLC plates (data not shown). For both ^99m^Tc-rCR2 and K41E ^99m^Tc-CR2 only one radioactive peak, with Rf = 0, was observed for the duration of the stability study for both PBS and serum (Table 1).

**Table 1:** Serum stability of technetium labeled rCR2 and K41E rCR2 protein.

		Incubation Time (hrs)					
		0.5	1	2	4	8	24
**rCR2**	**PBS**	96	97	95	96	98	96
	**Serum**	97	97	97	98	98	95
**K41E rCR2**	**PBS**	98	96	96	95	96	96
	**Serum**	97	96	96	97	97	96

## Discussion

Numerous human and animal studies have affirmed the link between IRI, complement activation and tissue damage, for example in myocardial infarction. Therapeutic agents that target C3 activation have reduced infarct size in animal models [29], [30]. In transplantation, IRI is unavoidable, especially when cadaveric donor organs are used. Greater IRI in cadaveric transplantation compared to living donors is believed to contribute to an increased incidence of delayed graft function (where dialysis is required for a period post transplantation) from 5% to 25% [31], [32]. A correlation between delayed graft function, increased rates of rejection and reduced long term graft survival and the length of the cold ischaemia time has also been established [2].

To our knowledge this is the first description of a radiolabeled imaging agent for the potential imaging of activated complement post ischemia reperfusion injury. Currently no techniques exist to non-invasively assess the degree of tissue injury within an affected organ soon after IRI. Most existing imaging techniques rely on functional or anatomical measurements such as organ perfusion or scar formation, respectively. These measurements are often done several days or even months post IRI and have little or no prognostic value. For example, the prevention of left ventricular (LV) dysfunction post-acute myocardial infarction (AMI) is currently addressed in the European Society of Cardiology and the European Association for Cardio-Thoracic Surgery guidelines by focusing on minimal treatment delays, aggressive reperfusion therapy, and the use of early and consistent optimal pharmacological therapy [33]. Recently, the MISSION! AMI clinical study [34] demonstrated that the highest incidence of arrhythmic death in post-AMI patients happens in the first three months after discharge in patients with normal LV ejection fraction who are thus considered at low risk. Several techniques have been proposed to stratify patients and indentify those with higher risk of sudden death following AMI. However, these techniques offered limited additional information and have a low positive predictive value (<30%) [34]. New methods are needed to solve the important unmet need of correct identification of high risk patients. One key aspect missing is a patho-physiological measurement of the injured tissue post IRI. Ideally a biomarker which can delineate the area at risk and the border zone around necrotic tissue soon after IRI, which also remains for several days, would be a powerful tool in risk stratification and guiding intervention. One potential biomarker which may meet these criteria is the activated complement molecule C3d. C3 is expressed in the local micro environment of stressed tissue soon after IRI which leads to the deposition of C3d on the plasma membrane of affected cells and can remain for several days.

To non-invasively evaluate C3d post IRI we engineered a recombinant complement receptor 2 with a C-terminal tag (VFPLECHHHHHH) designed for site-specific labeling with [^99m^Tc(CO)_3_]^+^. Under mild radiolabeling conditions (37°C for 30 min.), radiochemical yields exceeded 95% with a high specific activity of 10 MBq/ug. Therefore, with further optimization a simple kit-based labeling method could be developed without need for post-labeling purification. We previously reported that the addition of a CKLAAALEHHHHHH tag to the C-terminus of the phosphatidylserine (PS)-binding domain of synaptotagmin I (C2AcH), a cell death imaging agent, represented the first use of a combination of a His-tag and a free cysteine residue as a tag for incorporation of imaging probes into recombinant proteins. Although it was designed for versatility, to allow the incorporation of both a ^99m^Tc radiolabel via the His-tag and other imaging probes via covalent modification of the Cys, an unexpected benefit was dramatic increase in the efficiency of labeling with [^99m^Tc(CO)_3_]^+^, giving higher labeling yield and specific activity under mild conditions, compared with C2A with His-tag alone[17]. However for rCR2 we did not engineer a molecule without the C-terminal free Cys and so in this case we did not demonstrate that the combination of the Cys with the His-tag is responsible for the high radiochemical yield and high specific activity. Other groups have overcome this and shown that higher temperatures and/or longer incubation times are required to achieve adequate radiochemical yields with His-tagged proteins [18].

Recombinant human CR2 bound with high affinity (K_d_ = 500 nM) to human C3d enabling the detection of human and rat C3d deposited onto sheep RBC. The inactive K41E mutant of rCR2 did not bind to C3d and binding of rCR2 to human C3d was inhibited by anti-human C3d. We were also able to demonstrate that the Techenitum-99m radiolabeled rCR2 bound specifically to C3d^+^ RBCs. This binding was inhibited by anti-C3d. The inactive mutant K41E CR2 and the irrelevant protein C2A radiolabeled comparably to rCR2 but did not bind to C3d. It is of particular importance in this case not to have used an “amine directed” non-specific bifunctional chelator for radiolabeling rCR2, as there is a greater likelihood of modifying Lysine residue 41 in CR2 and rendering it unable to bind to C3d. Such an approach was recently used to non-specifically cross link recombinant CR2 fragment to superparamagnetic iron oxide (SPIO) nanoparticles to generate targeted MR contrast agent for imaging renal inflammation [35]. The affinity of ^99m^Tc-CR2 was also measure by ELISA and gave rise to K_d_ of ∼25 nM. This affinity is lower than observed by surface plasma resonance and be due to using different binding buffers. It has previously been reported that the binding of CR2 to C3d is dependent on the buffer salt concentration [28].

In conclusion, we have developed and characterized a new and versatile radiopharmaceutical for the potential of imaging activated complement, based on rCR2, which contains the C3d binding domain of complement receptor 2. It site-specifically incorporates [^99m^Tc(CO)_3_]^+^ with excellent efficiency and is stable in serum. The new site-specifically labeled rCR2-^99m^Tc has excellent affinity for C3d, and warrants *in vivo* evaluation for imaging activated complement post ischemia reperfusion injury in cardiovascular and graft rejection preclinical models.
